# Dynamic Voltage Frequency Scaling Simulator for Real Workflows Energy-Aware Management in Green Cloud Computing

**DOI:** 10.1371/journal.pone.0169803

**Published:** 2017-01-13

**Authors:** Iván Tomás Cotes-Ruiz, Rocío P. Prado, Sebastián García-Galán, José Enrique Muñoz-Expósito, Nicolás Ruiz-Reyes

**Affiliations:** Telecommunication Engineering Department, University of Jaén, Science and Technology Campus, Linares, Spain; West Virginia University, UNITED STATES

## Abstract

Nowadays, the growing computational capabilities of Cloud systems rely on the reduction of the consumed power of their data centers to make them sustainable and economically profitable. The efficient management of computing resources is at the heart of any energy-aware data center and of special relevance is the adaptation of its performance to workload. Intensive computing applications in diverse areas of science generate complex workload called workflows, whose successful management in terms of energy saving is still at its beginning. WorkflowSim is currently one of the most advanced simulators for research on workflows processing, offering advanced features such as task clustering and failure policies. In this work, an expected power-aware extension of WorkflowSim is presented. This new tool integrates a power model based on a computing-plus-communication design to allow the optimization of new management strategies in energy saving considering computing, reconfiguration and networks costs as well as quality of service, and it incorporates the preeminent strategy for on host energy saving: Dynamic Voltage Frequency Scaling (DVFS). The simulator is designed to be consistent in different real scenarios and to include a wide repertory of DVFS governors. Results showing the validity of the simulator in terms of resources utilization, frequency and voltage scaling, power, energy and time saving are presented. Also, results achieved by the intra-host DVFS strategy with different governors are compared to those of the data center using a recent and successful DVFS-based inter-host scheduling strategy as overlapped mechanism to the DVFS intra-host technique.

## Introduction

Cloud Computing is a distributed processing paradigm leading the next generation computational platforms based on the externalization of computing needs offered as services [1]. Data centers are the backend computing infrastructures that make up Cloud systems whose performance have a significant economic and environmental impact. It is estimated that data centers consume around 1.5% of the world´s electricity and they are responsible for the emission of about 2% of the CO_2_ worldwide with energy costs in an average data center doubling every five years [2, 3]. Designing faster and more powerful data centers has been a priority in the information technology (IT) industry in the recent years. To this end, the number of resources working collaboratively has been increased and the associated processing capabilities have been improved, what has lead to an increment in the demanded power. Also, data centers need the support of auxiliary infrastructures such as cooling systems, variable-speeds drivers, temperature and humidity sets and power distribution units to maintain the servers farms. Furthermore, the storage, power distribution and cooling systems are generally over provisioned in order to ensure the reliability, what also increases energy consumption [4]. Hence, the crescent number of servers and auxiliary systems in data centers makes their overall performance cost and environmental impact to increase non-stop as these services are more and more demanded currently [5–7]. In this scenario, making energy saving a critical aspect to be aware of in the whole management of the Cloud systems is necessary to allow their growth in terms of sustainability and economic benefit.

Green Cloud Computing is intended to provide users with the same quality of service requirements as Cloud Computing but lowering data centers power consumption through a more efficient use of their resources [3]. The efficiency of data centers generally falls into four main types: power infrastructure, cooling, airflow management and IT efficiency [8, 9]. Improving data centers through IT involves using more adequate servers, networks and data storage resources as well as managing these servers, networks and storage systems more efficiently. Particularly, job scheduling is at the core of the successful power management in Cloud Computing and there is a main strategy for reducing energy consumption: Dynamic Voltage and Frequency Scaling (DVFS), which allows to dynamically adapt the machines performance to the changing conditions of the workload [2]. DVFS technique cannot only be applied for intra-host energy saving but for inter-host energy saving, such as in the recent and successfull DVFS-based scheduling mechanims described in [10–13]. The application of DVFS is being studied in sequential workload execution. However, its application and analysis in other industrial, business and scientific applications of Cloud Computing is still in an initial stage, such as the case of workflows or workload where the different jobs are interrelated and complex dependencies must be considered.

The computation of workflows extracted from large scale data and computation intensive applications is extensively used by prestigious scientific institutions nowadays in many science and technology fields [14, 15]. The bioinformatics project at Harvard University is a relevant example, which conducts a wide search for small untranslated RNAs (sRNAs) that rule diverse processes such as secretion or virulence in bacteria [16]. Also, it is worth mentioning the applications of workflows in astronomy such as Montage engine, promoted by NASA’s Earth Sciences Technology Office to generate science mosaics combining diverse images from the sky [17]. A current challenge is how to manage these workflows in Cloud Computing in an efficient way in the sense of energy consumption and new strategies are being proposed. Conducting experiments with real data centers implies several difficulties. On the one hand, a considerable investment must be done to deploy the real computational infrastructures associated to Cloud systems and on the other hand, the energy associated costs to run extensive workflow applications, which may make prohibitive the research for the major part of institutions. Simulators allow to perform experiments with real scenarios both in terms of supporting infrastructures as well as of workload without the need for any specific physical equipment or networks. This way, the availability of sophisticated simulators is relevant in the study of new green strategies for management in Cloud Computing [18].

In this work, a new Cloud Computing simulator is proposed: WorkflowSim-DVFS. This software tool is an expected energy-aware extension of the WorkflowSim simulator based on Java [19, 20], which incorporates the Dynamic Voltage Frequency Scaling technique [21–23] in a way that real workflows can be processed considering a regulation of the voltage and frequency in the computing resources as done in current CPUs. It must be underlined that WorkflowSim is the only Cloud Computing simulator nowadays that allows the execution of real workflows considering the event of failures and different amount of workload in each server [19]. Now, this work allows this simulator to be energy-aware providing a computing-plus-communication power model that considers computing, reconfiguration and network costs [10–13] and incorporating five different DVFS governors. To validate the simulator, experiments are conducted in different real scenarios and performance is discussed considering both the results of the DVFS intra-host governors and the results of a recent DVFS-based inter-host scheduling strategy used as an overlapped mechanism to the DVFS intra-host governors to increase energy saving [10–13]. The simulator is offered as an open source tool and it has been made available from the GitHub repository [24]. Hence, this work represents a further effort to support research on Green Cloud Computing systems.

This paper is organized as follows. First, in “Material and Methods” the definition of the problem and the related works in the field of power-aware and workflows simulators in Cloud Computing are introduced followed by the motivation of this work. Next, the proposed simulator allowing the energy analysis and optimization as well as the DVFS management of workflows in Cloud Computing is presented in this section. In “Experimental Evaluation and Discussion” experimental results considering diverse scenarios are analyzed to evaluate the simulator. Governors are tested when used uniquely as intra-host strategies for energy saving and when they are also used in inter-host DVFS-based scheduling strategies. Finally, the main conclusions of the work are drawn in “Conclusions”.

## Materials and Methods

### Definition of the Problem

The problem in hand in this work is to offer an easy, realistic and open source simulator to help research on energy optimization in Clouds running workflows. The specific aspects of this problem can be summarized as follows:

1. The simulator must provide DVFS capabilities controled by the main types of governors to Cloud data centers. DVFS [2] is a key strategy for energy optimization in Cloud Computing, as shown by the current emergence of many energy optimization strategies that are being developed considering DVFS-enabled networked data centers (e.g., jobs scheduling [10–13]). In essence, DVFS is a technique that, based on the type of governor, adjusts frequency levels of processing elements according to the amount of workload to be executed. This is translated in an adjustable power capability for these systems. High performance of the processors, generally measured in millions of instructions per second (MIPS), are obtained using high powers and used at the event of high levels of workload, whereas low performance can be provided in low workload states with the associated power reduction. DVFS is included in many current computing systems such as the Linux kernel and it is generally not considered a substitute of other strategies for energy optimization, but a background intra-host tool supporting the design of new strategies for energy saving in data centers. The simulator must include the main different types of DVFS governors which can be classified into static and dynamic.
2. The second main aspect of the problem is to offer a simulator able to estimate the energy, and related parameters, of the Cloud. Once the execution of the workload has finished, it must offer an estimation of the overall and average power and consumed energy as well as of makespan. If a simulator cannot provide an estimation of these parameters, it is not possible to inform the Cloud administrator or user about the performance of the designed system and in this way, the strategies for energy saving cannot be analyzed and improved. To solve this problem, it is necessary to adopt a realistic model and integrate it in the simulator. Furthermore, it is relevant that the power model does not only consider the computing cost of the processing elements but also, the reconfiguration consumption in dynamic DVFS governors and the network costs, as recent and successful works in Cloud Computing propose [10–13].
3. The simulator must be able to simulate traces that represent a real processing in Clouds. If the tasks of the workload are randomly generated, the execution of these tasks in the simulator does not correspond to a real execution of workload in a Cloud data center and thus, the performance of the systems indicated by the simulator would not be close to real-world results in many problems. In this work, the simulation of workflows is considered. The ability to process workflows is useful to simulate traces that reproduce real workload in many real systems. Workflows determine the order in which tasks must be processed, including constrains that avoid that tasks are processed before their parent tasks, and the inputs and outputs results that must be considered for each task. This assures that tasks are processed following a predefined order with the associated input and output. Furthermore, the simulator must be able to execute real traces instead of series of random tasks, what helps in providing a realistic tool for optimization in Cloud environments.
4. Also, the simulator must offer task clustering, events of tasks failures and overheads and policies for the management of these failures. Hence, the realism of the simulator must not only be based on power capabilities for optimization and processing of real workloads but also, advanced techniques in Cloud Computing for the network management must be integrated.
5. Finally, the simulator must be open source software. It is intended that the simulator constitutes a free available tool for the research community, in a way that further improvements in energy saving through the design of new strategies based on DVFS-enabled networks can be achieved more easily. Also, this simulator could be enhanced by researchers in different topics of Cloud management (e.g., security or interconnection among Clouds).

In the next section, the different types of power-aware simulators for Cloud Computing are analyzed and discussed to finally present the motivations of the suggested proposal in this work.

### Related Works and Motivation of the Proposal

In the last years the development of simulators for Cloud Computing has experienced a great evolution in a parallel way to the expansion of Cloud systems in both industrial and scientific fields. Particularly, in this section it is important to study those simulators that present capabilities related to workflow management and energy efficiency.

In [25], B. Aksanli et al. present a comparative study of data centers simulators supporting energy simulations to finally focus on the GENSim simulator, the only data centers simulator able to estimate the impact of the allocation of services and batch jobs on a single server. Results in this work demonstrate that green energy forecasting can improve overall energetic efficiency in a data center. However, this simulator does not support simulations of Virtual Machines (VMs), it does not implement DVFS and it does not support the execution of workflows.

The work presented in [26] performs a comparison between existing Cloud simulators and it proposes the GreenCloud simulator. GreenCloud is an extension of the well-known ns-2 network simulator, able to offer details of energy consumption in networks links, routers and servers and including two strategies for on host energy saving: Dynamic Voltage Scaling (DVS) that allows decreasing voltage of switches and Dynamic Network Shutdown (DNS) to perform an ON/OFF strategy. Also, it offers an accurate analysis of communications at the TCP level. However, this simulator is not an open source tool and thus, the accessibility for researchers and the flexibility to introduce new strategies for the Cloud management decreases significantly [19].

CloudSim [18] is an open source Cloud Computing simulator written on Java that allows modeling Cloud infrastructure and services supporting the execution of sequential workloads. It provides a simple model of execution of tasks (no possibility of workflows simulation) and it does not consider the dependence among these tasks or task clustering. Also, it does not contemplate the occurrence of faults or overheads. Thus, this simulator is based on some simplifications that do not conform to the actual dynamic environment of distributed Cloud systems and to the evolution of new management techniques for workflows. Furthermore, it does not offer energy-aware capabilities such as DVFS or DNS.

W. Chen and E. Deelman [27] presented an extension of CloudSim called WorkflowSim that allows the execution of workflows. WorkflowSim implements a layer for managing execution of workloads with dependencies. It also offers other features such as the grouping of tasks or clustering where small tasks on large jobs are linked to reduce overloads. Further, it allows experiments with real workflows described in DAX format or Directed Acyclic Graphs (DAGs) in XML format [28], used by the Pegasus system [28] workflows in a way that new scheduling algorithms can be validated in realistic scenarios. WorkflowSim also allows mechanisms for fault simulation in task execution and moreover, it implements policies for the management of these failures. However, it does not have any characteristics for energy saving or analysis.

T. Guérout et al. [29] extended CloudSim to implement DVFS into CloudSim and to include tools to estimate power and energy consumption. Also, the simulator considers the execution of workflows modeled as DAGs. In the same way, F. Cao et al. [30] also propose a simulator including DAG workflows and DVFS to obtain the optimum frequency without mentioning the implemented governors and associated possible configurations and no source code is made available. However, in both previous works it cannot be assured that the obtained simulations can describe a behavior alike a real data center since they do not incorporate fault simulation in tasks execution and overheads, which are essential to accurately simulate workload execution in the ever-changing computing environment of Clouds. Furthermore, the power model only considers the computing cost of the processing elements and it is not aware of the reconfiguration of frequencies and network costs. Thereby, many recent DVFS-based scheduling strategies cannot be implemented in these simulators [10–13].

After studying the current available Cloud simulators above, it is appreciated that there does not exist a platform that meets all the requirements presented in the problem definition. However, WorkflowSim, even though it does not cover all the requirements, could offer a whole solution to the problem if it could integrate DVFS governors and a power model considering the computing, reconfiguration of frequencies and network costs. As introduced before, WorkflowSim is an open source simulator able to process workflows and providing capabilities for task clustering, event of tasks failure and overheads and policies for management of these failures and so, the incorporation of DVFS governors and a computing-plus-communication power model could make of it a complete solution to the problem. Hence, it is proposed in this work to extend WorkflowSim to become power-aware and integrate DVFS models that have been experimentally validated in real hosts [29]. This offers a realistic and open source tool for further research on Green Cloud Computing in which energy consumption can be estimated and the experiments are based on real workflows. Moreover, the simulator offers a platform for research on DVFS-enabled data centers. Since modern CPUs already include DVFS techniques, it is important that the simulator can include this technique so that the CPUs dynamic power changes can be considered in the whole energy estimation and implementation of DVFS-based optimization strategies as in real world. In this way, with WorkflowSim-DVFS, recent and successfull DVFS-based scheduling algorithms [10–13] can be simulated and optimized based on the power consumption estimation of computing, reconfiguration and network costs, at the same time task clustering, event of tasks failure and overheads and policies for management of these failures are also offered. Hence, energy-aware capabilities are introduced to one of the most realistic Cloud simulators nowadays and the code is made available for the research community. This work represents an expected new effort to offer accurate energy-aware simulators of real workflows in Cloud Computing [19].

### Proposed Energy-Aware Simulator for Real Workflows Management: WorkflowSim-DVFS

The proposed simulator includes the DVFS technique with diverse governors as in modern CPUs nowadays, and it is able to estimate the power consumption of the processing of real workflows. The incorporation of these features in WorkflowSim is explained in this section.

#### DVFS for real workflows processing and computing cost model

The methods for energy saving in data centers can be classified into host (or intra-host) and network (or inter-host) levels. Whereas the network level energy-aware methods are essentially founded on the coordination and cooperation of machines through scheduling processes to reduce power consumption, the host level ones are devoted to increase inside-machine efficiency. This work focuses on the study and implementation of the host energy-aware DVFS technique for real Cloud simulators. DVFS offers an efficient and gradual way to reduce the dissipated power in a processor by adjusting its clock speed and the supplied voltage during both phases of idleness and intensive computing in applications execution. Using this technique large reductions in power consumption with a very slight loss of efficiency in its performance are achieved. Different high performance computing platforms based on data centers such as cluster computing and supercomputing apply DVFS technologies to reduce power consumption and to get a high reliability and availability in their infrastructures [21–23]. Furthermore, currently the CPUs market provides such technologies, as is the case of Intel’s SpeedStep and AMD’s PowerNow!. These CPUs are able to dynamically vary their voltage and frequency to adapt to the workload, mainly trying to reduce energy consumption but, generally, also avoiding a delay in the execution.

In the Linux kernel, DVFS can be ruled by different types of governors. Governors are core models that can manage the operation points both in frequency and voltage based on an algorithm. Currently, there exist five types of DVFS governors:

- Performance: it sets the frequency in a static way to the greater available CPU frequency.
- Powersave: it sets the frequency in a static way to the lowest available CPU frequency.
- Userspace: it sets frequency according to a user program.
- On demand: it makes an adjustment of frequency based on the utilization of the CPU and a predefined utilization threshold.
- Conservative: it is a conservative approach of the previous On demand governor. It also makes an adjustment of frequency based on the utilization of the CPU, but in a more gradual way.

While basic governors such as Performance and Powersave use a fixed frequency, more complex governors are based on thresholds and voltage and frequency are varied based on the current utilization respect to the configured threshold. This is the case of On demand and Conservative governors, where voltage and frequency are reduced when the utilization is low and increased when the utilization is high, what is translated into energy saving.

In DVFS, the dependence of the computing power of a processing element with voltage and frequency is given by the following expression [31]:

$$\begin{array}{c} P\left(V,f\right)=aCV^{2}f \end{array}$$ (1)

where *V* represents the voltage, *f* indicates the frequency and *a* and *C* are constants or frequency multipliers. A CPU can work at a certain number of different frequencies depending on the multipliers *a* and *C*, and voltage is scaled with the frequency. Lower voltage implies that lower frequencies can be selected on the CPU, as both parameters are interrelated. From this expression it can be derived that the more voltage and frequency decrease, the less computing power is consumed.

In order to analyze the performance of DVFS in simulators in terms of power saving, a computing power model that estimates the power consumption based on a real CPUs behavior is necessary. High-level approaches increase the portability and speed in simulations in contrast to more complex models that consider low-level aspects of the computing platform and offer a more accurate CPU computing cost. In this way, for simulation, high-level models are generally considered. Following the approach of T. Guérout et al. [29], the overall computing power consumed by the CPU, *P*_*total*_(*α*, *V*, *f*), is given by a linear relation of the CPU’s utilization rate (*α*) and the associated power consumption of the CPU when *α* = 0 and *α* = 1, *P*_*idle*_(*V*, *f*) and *P*_*full*_(*V*, *f*), respectively:

$$\begin{array}{c} P_{total}\left(\alpha ,V,f\right)=P_{idle}\left(V,f\right)+\left[P_{full}\left(V,f\right)-P_{idle}\left(V,f\right)\right]\alpha \end{array}$$ (2)

where *α* is the utilization rate of the CPU, a real number in the range [0, 1], being *α* = 0 the case when the machine is in the lowest performance state (i.e., idle state) and the associated power consumption is *P*_*idle*_(*V*, *f*), whereas *α* = 1 is the case when the machine is in the highest performance state (i.e., full state) during the processing of workload and the associated power consumption is *P*_*full*_(*V*, *f*). Considering the relation of frequency *f* and voltage *V* of a CPU with multipliers *C* and *a* introduced in Eq 1, *P*_*idle*_(*V*, *f*) and *P*_*full*_(*V*, *f*) can be calculated. On the one hand, for the idle state (i.e., *α* = 0) the power consumption *P*_*idle*_(*V*, *f*) can be expressed as:

$$\begin{array}{c} P\left(0,V,f\right)=P_{idle}\left(V,f\right)=aCV_{idle}^{2}f_{idle} \end{array}$$ (3)

where *V*_*idle*_ and *f*_*idle*_ denote the voltage *V* and frequency *f* when the CPU is in the idle state, respectively. On the other hand, the expression for power in the full state (i.e., *α* = 1) *P*_*full*_(*V*, *f*) corresponds to:

$$\begin{array}{c} P\left(1,V,f\right)=P_{full}\left(V,f\right)=aCV_{full}^{2}f_{full} \end{array}$$ (4)

where *V*_*full*_ and *f*_*full*_ denote the voltage *V* and frequency *f* when the CPU is in the full state, respectively. The utilization rate *α* is also used in DVFS to check the thresholds of the considered governors and it represents the scale in frequency *f* and voltage *V* in Eq 1. The integration of DVFS in WorkflowSim is relevant to allow on host energy optimization through CPU’s dynamic performance.

#### WorkflowSim-DVFS entities fundamentals

**Fig 1:**
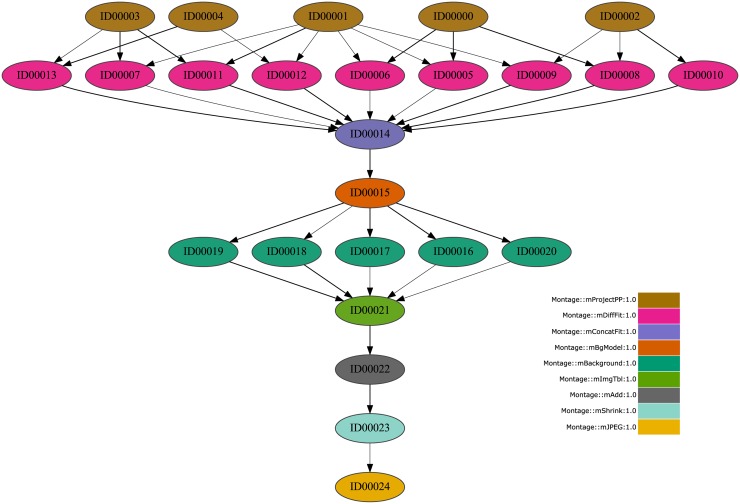
Montage Workflow DAX (25 jobs) example.

**Fig 2:**
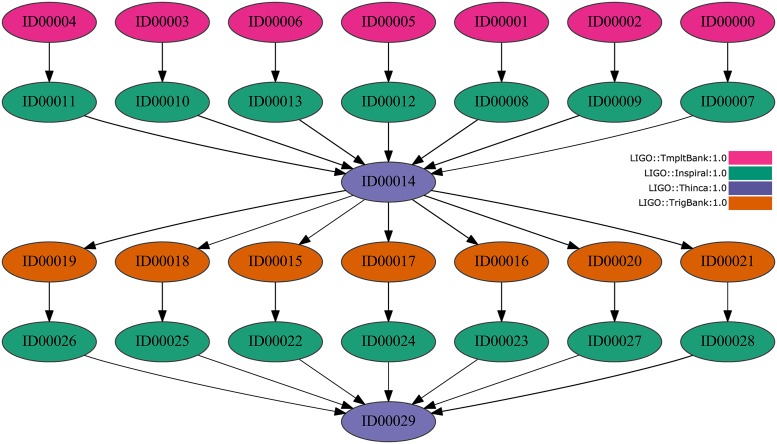
Inspiral Workflow DAX (30 jobs) example.

**Fig 3:**
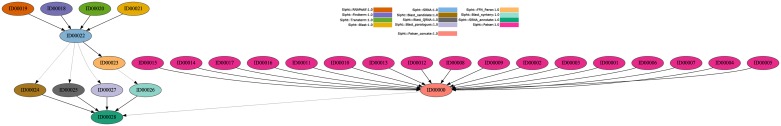
Sipht Workflow DAX (30 jobs) example.

WorkflowSim extends CloudSim to process DAXs workflows or workloads where tasks have complex dependencies. As an example, Figs 1, 2 and 3 graphically show three workflows corresponding to real traces from Montage (25 jobs), Inspiral (30 jobs) and Sipht (30 jobs) projects [16], respectively, where the type of jobs and dependences can be observed.

**Fig 4:**
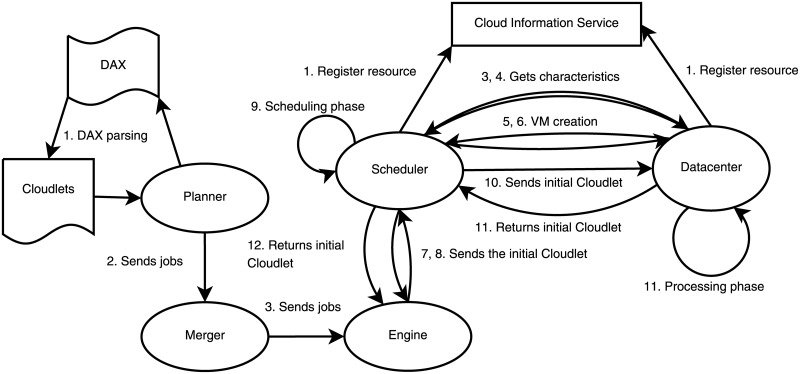
WorkflowSim messages—Initialization stage.

**Fig 5:**
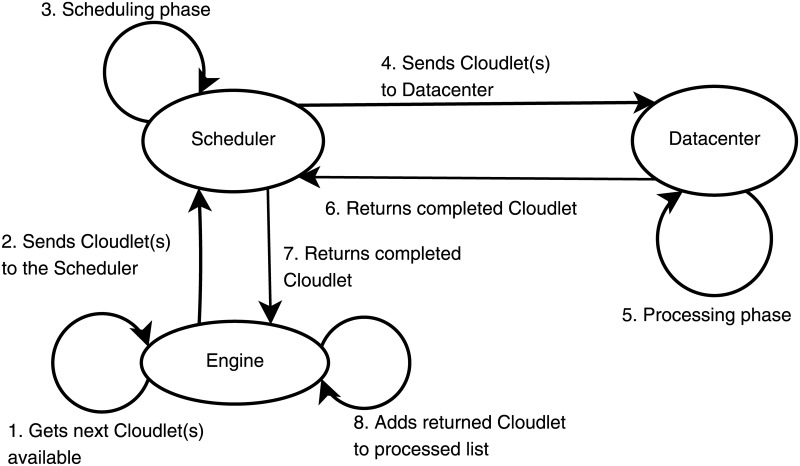
WorkflowSim messages—Main stage.

**Fig 6:**
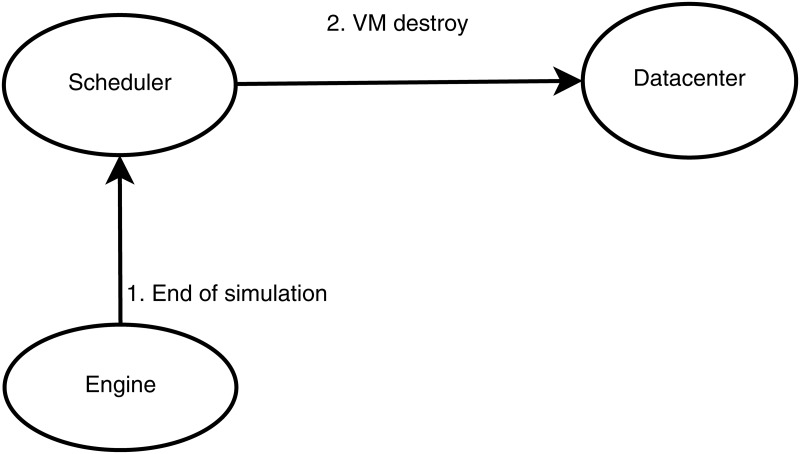
WorkflowSim messages—Ending stage.

Five main entities conduct the management of the workload in WorkflowsSim-DVFS: the Planner, the Merger, the Engine, the Scheduler and the Datacenter. The Planner is the entity that initializes the system and parses the DAX file to get the individuals tasks called Cloudlets. Then, those tasks are sent to the Merger (also called Clustering Engine) that groups the different tasks into jobs (i.e., collection of tasks). The default configuration of the simulator leaves no clustering performed, it just gets each task into a job individually. Next, the jobs are sent to the Engine, where they are selected following the order specified by the workflow. The Engine is the entity in charge of making sure the workflow’s order is followed, and sending to the Scheduler the tasks that can be processed each time a task is returned, being these tasks those whose parent nodes have already been processed. Finally, the Scheduler selects which VM of the Datacenter is the most suitable for processing each task. Communications are grouped in three stages: Initialization stage, Main stage and Ending stage. Figs 4, 5 and 6 graphically represent the messages exchange between these entities in each stage, respectively.

The process for the main stage is repeated each time the Datacenter entity finishes processing a task. It must be noted that to make this execution close to the behavior of a real and power-aware data center using workflows, the Scheduler must not only be able to use information related to execution times but also, power and energy parameters, and furthermore, the hosts within the Datacenter must include DVFS govenors.

#### Computing-plus-communication power model for real workflows processing

The integrated computing power model in our simulator describes how power varies with frequency in a real processor with DVFS following the approach in [29] as introduced in the previous section. In this computing power model (Eq 2) consumed power of the processing elements varies depending on frequency, and the values of frequency that a processor can work with are obtained multiplying the mother board’s base frequency by the processor’s frequency multiplier, so that the different available frequency values are discrete. Table 1 presents a real example of the possible frequencies and associated frequency multipliers of a real processor (CPU Intel(R) Core(TM)2 Quad CPU Q6700 @ 2.66GHz with 4GB Ram).

**Table 1:** DVFS parameters of a CPU Intel(R) Core(TM)2 Quad CPU Q6700 @ 2.66GHz with 4GB Ram for the computing power model: Frequency, frequency index, base frequency multiplier, performance in MIPS and power in full and idle states for the corresponding frequency.

**Frequency (GHz)**	1.600	1.867	2.113	2.400	2.670
**Frequency index**	0	1	2	3	4
**Base frequency (2.670 GHz) multiplier**	59.925	69.93	79.89	89.89	100
**Performance (MIPS)**	898.875	1048.95	1198.35	1348.35	1500
**Null utilization, *P*_*idle*_ (W)**	82.75	82.85	85.95	83.10	83.25
**Full utilization, *P*_*full*_ (W)**	88.77	92.00	95.5	99.45	103.0

As shown in Table 1, the possible discrete frequencies for this processor are 2.670 GHz, 2.400 GHz, 2.113 GHz, 1.867 GHz and 1.600 GHz, which are the corresponding values of the multiplication of the base frequency (2.670 GHz) by the base frequency multipliers (i.e., 100, 89.89, 79.89, 69.93 and 59.875, respectively). Also, as it can be observed in Table 1, each frequency is associated to a performance value in MIPS for the processing element. Specifically, the proposed computing model (Eq 2) depends on the required power of the processing element in the full and idle states, *P*_*full*_ and *P*_*idle*_, respectively, for the corresponding working frequency. Finally, Eq 2 depends on the utilization rate *α*. Hence, dynamic DVFS governors must select the most convenient frequency on their criteria to execute workload and so forth, the corresponding performance in term of MIPS and the associated *P*_*full*_ and *P*_*idle*_ values. In this way, dynamic governors are responsible for comparing the current utilization *α* to predefined thresholds of utilization to know whether the processing element needs higher performance (MIPS) or, on the contrary, the processor is rather idle and it can be scaled down to save unused power. For instance, let a certain processing element in the simulator to be working at frequency 2.400 GHz (frequency index 3 and base frequency multiplier 89.89) and consequently, the processing element performance in terms of MIPS is 1348.35, *P*_*full*_ = 99.45*W* and *P*_*idle*_ = 83.10*W*. When the utilization of machine *α* is checked, it is observed that its current value is 0.8. Hence, the current computing power of the processing element can be calculated, following Eq 2, as:

$$\begin{array}{c} P_{total}\left(\alpha ,V,f\right)=P_{idle}\left(V,f\right)+\left[P_{full}\left(V,f\right)-P_{idle}\left(V,f\right)\right]\alpha =83.10W+\left[99.45W-83.10W\right]0.8=96.18W \end{array}$$ (5)

In the simulations in this work, as proposed in [29], the type of processing elements used is CPU Intel(R) Core(TM)2 Quad CPU Q6700 @ 2.66GHz with 4GB Ram. The power values are measured using a plogg wireless electricity meter. Of course, power consumption can be measured in the physical processor, but in the simulator, this power model works with linear interpolation based on the real values *P*_*full*_ and *P*_*idle*_ for each frequency, estimating the power depending on the utilization rate *α* as indicated in Eq 2.

Finally, the computing-plus-communication power model of the simulator adds the reconfiguration of frequencies and network costs to the studied computing cost as proposed in [10–13]. The specific values for the experimental evaluation are introduced in “Experimental Evaluation and Discussion”.

#### Entities communications for workflows processing

The entities messages in the proposed simulator are not modified from the previous simulator WorkflowSim. However, since the proposed simulator is power aware, the steps involving Tag 41 change considerably. Tag 41 is the simulator state message that appears at the end of the initialization and main stages in WorkflowSim-DVFS. The appearance of this message means that, if the selected governor for the processing elements is dynamic (i.e., OnDemand or Conservative), the utilization of the processing element must be compared to the predefined utilization threshold of the governor in order to adapt its frequency and so forth, its performance in terms of MIPS to the current workload conditions. This update process is performed in a recurrent way every time Tag 41 state message appears in the system. The checking interval parameter set in the Datacenter creation is the lapse of time between these messages. The default value for the checking interval is 0.01 s. Thus, each 0.01 s the simulator throws a Tag 41 message. This indicates how often the Datacenter checks the utilization of processing elements with dynamic governors and it applies the DVFS algorithm to determine whether the Datacenter is idle and the processing elements performance must be scaled down to save energy or, on the contrary, the utilization of the processing elements is too high that surpasses by excess the threshold and the system needs to be scaled up to increase its performance. For instance, for a Cloudlet being processed for 11 s, the number of messages with Tag 41 that the simulator produces is 1100, considering the default value of the checking interval set to 0.01 s. Similarly to WorkflowSim, the main stage process is repeated each time the Datacenter has finished processing a Cloudlet. The Engine then checks whether some new Cloudlets could be executed, it sends them to the Scheduler which in turn, takes decision related to VMs and sends them to be processed. Whenever the simulation receives a Tag 41 message, the governors of the processing elements check (for those using dynamic governors) the relation between the utilization threshold and the utilization of the processing elements. In this step, also the power consumption in the simulator is updated.

Finally, in Algorithm 1, the performance of the proposed simulator WorkflowSim-DVFS is formally presented using pseudo-code and each step is explained. For further details, the full code is available in [24].

**Algorithm 1** WorkflowSim-DVFS pseudo-code.

1: —Data

2: DAX with Workflow Jobs   ⊳*Workflow associated file considering Jobs characteristics and dependences*

3:

4: **** **Initialization Stage** ****

5:

6: Generate: Planner, Merger, Engine, Scheduler, Datacenter, Cloud Information System (CIS)   ⊳*Generate main entities*

7: Jobs = DAXParsing   ⊳*DAX is stored in the Planner in a Jobs list considering their characteristics and dependences*

8: Datacenter.DatacenterCharacteristics.TypeofGovernor = DVFSStrategySelection   ⊳*A DVFS governor must be chosen for the processing elements: Performance, PowerSave, UserSpace, On Demand or Conservative*

9: RegisterResourcesInCIS(Datacenter, Scheduler, CIS)   ⊳*The characteristics of the Datacenter and Scheduler are registered in the CIS*

10: SendJobs (Planner, Merger, Jobs)   ⊳*The Planner sends to the Merger the whole list of jobs to be processed*

11: SendJobs (Merger, Engine, Jobs)   ⊳*The Merger sends to the Engine the whole list of jobs to be processed*

12: Datacenter.DatacenterCharacteristics = GetCharacteristics (Scheduler, Datacenter)   ⊳*The Scheduler must be aware of the characteristics of Datacenter resources*

13: VMlist = VMCreation (Scheduler, Datacenter)   ⊳*The Scheduler creates VMs in the resources of the Datacenter*

14: SendJobs (Engine, Scheduler, InitializationJob)   ⊳*The Engine sends an initial job which does not belong to the DAX to generate an initial state*

15: SelectedVM = Schedule (Scheduler, VMlist, InitializationJob)   ⊳*The Scheduler decides the VM to process the initial job*

16: ProcessJob (Scheduler, SelectedVM, InitializationJob)   ⊳*The initialization job is scheduled to the selected VM*

17: **if** Governor == OnDemand or Conservative **then**   ⊳*If the selected DVFS governor is dynamic (Tag 41)*

18:  **while** InitializationJob is Being Processed **do**   ⊳*Every 0.01s*

19:   DVFSPowerCheck   ⊳*The utilization of the involved processing element must be checked*

20:   **if** SelectedVM.CPUPerformanceToBeUpdated **then**   ⊳*If there is need to increase o decrease the power of the processing element to increase or decrease the associated performace (MIPS)*

21:    SelectedVM.CPU.ModifyFrequencyMultiplier   ⊳*The frequency of the governor must be scaled accordingly*

22:   **end if**

23:  **end while**

24: **end if**

25: ReturnJobs (Scheduler, Engine, InitializationJob)   ⊳*The processing of the initialization job has finished*

26:

27: **** **Main Stage** ****

28:

29: **for** k < NumberOfJobsRows; k++ **do**   ⊳*Process all the remaining Jobs considering full rows of Jobs of the workflow*

30:  JobsRow = GetNextJobsRow (Engine, Jobs)   ⊳*Jobs within a row are in the same level in the workflow and have no dependences among them*

31:  SendJobsRow (Engine, Scheduler, JobsRow)   ⊳*The Engine sends a set of Jobs to the scheduler*

32:  SelectedVMs = Schedule (Scheduler, VMlist, JobsRow)   ⊳*The Scheduler selects the VMs to process these Jobs*

33:  ProcessJobsRow (Scheduler, SelectedVMs, JobsRow)   ⊳*The Jobs are sent to the selected VMs to be processed*

34:  **if** Governor == OnDemand or Conservative **then**   ⊳*If the selected DVFS governor is dynamic (Tag 41)*

35:   **while** JobsRow is being processed **do**   ⊳*Every 0.01s*

36:    DVFSPowerCheck   ⊳*The utilization of the involved processing elements must be checked*

37:    **if** SelectedVMs.CPUPerformanceToBeUpdated **then**   ⊳*If there is need to increase o decrease the power of any processing element to increase or decrease the associated performace (MIPS)*

38:     SelectedVMs.CPU.ModifyFrequencyMultiplier   ⊳*The frequency of every governor must be scaled accordingly*

39:    **end if**

40:   **end while**

41:  **end if**

42:  CheckAndSolveFailuresEvents   ⊳*Check failures policies and solve them if any*

43:  ProcessedJobs++   ⊳*The number of processed Jobs must be incremented*

44:  UpdateEnergyPowerAndTimeResults   ⊳*Energy and power must be updated accordingly to the power model. Processing time is also updated*.

45:  ReturnJobs (Scheduler, Engine, JobsRow)   ⊳*The processing of the set of Jobs has finished*

46: **end for**

47:

48: **** **End Stage** ****

49:

50: EndSimulation (Engine, Scheduler)   ⊳*The Engine informs the Scheduler that the whole DAX has been executed and so the simulation has finished*

51: VMdestroy (Scheduler, Datacenter)   ⊳*VMs are destroyed*

52: ProvideFinalEnergyPowerAndTimeResults   ⊳*Final results in energy, power and time of the workflow processing are provided*

## Experimental Evaluation and Discussion

In this section, the validation of the proposed simulator in diverse scenarios is presented. Performance is analyzed in terms of time, power and energy and results are compared and discussed for different governors in DVFS. Also, the evolution of CPU utilization, frequency indexes and computing power along workflows processing are studied.

### Scenarios

The simulator has been tested using twelve different real workflows, from Montage, Inspiral and Sipht scientific projects [16, 32]. The characteristics of these workflows and considered network topology to test the simulator are presented as follows.

#### Workload description

**Montage workflows**: As introduced before, Montage [16, 32] is an open source toolkit developed by the NASA/IPAC Infrared Science Archive to create custom mosaics of the sky in astronomy through input images in FITS format. The number of jobs that are involved in a Montage workflow is a function of the number of input images to make up the final mosaic of the sky area in a way that, the more retrieved images, the more involved jobs. Fig 1 represents a Montage workflow of 25 jobs. It can be observed 9 sorts of jobs related to a level of execution. Hence, mProjectPP jobs correspond to processing level 1 and mJPEG jobs correspond to processing level 9. In this work traces from Montage project with 25, 50, 100 and 1000 jobs are considered in a way that the simulator is evaluated in the conditions of low, medium and high load.

**Inspiral workflows**: Inspiral workflows are retrieved from The Laser Interferometer Gravitational Wave Observatory (LIGO) [16, 32]. The aim of this observatory is to identify gravitational waves originated by diverse phenomena in the universe on the basis of Einstein’s theory of general relativity. Specifically, the LIGO Inspiral workflows are applied to study the data collected from the combination of compact binary systems (e.g., binary neutron stars and black holes) and they are made up of four different types of jobs. An example of an Inspiral workflow structure involving 30 jobs can be observed in Fig 2. To test the proposed simulator, four different traces with 30, 50, 100 and 1000 jobs are processed.

**Sipht workflows**: The origin of Sipht workflows lies in a bioinformatics project conducted at Harvard University to found small untranslated RNAs or sRNA that rule diverse processes as secretion or virulence in bacteria. In order to help sRNA search, the high-throughput technology SIPHT program was developed [16, 32]. To be precise, this program helps to automatize the identification of sRNA encoding genes for the bacterial samples in the National Center for Biotechnology Information database. Sipht workflows consist of 13 types of jobs as shown in Fig 3. As in the previous cases, four traces grouping 30, 60, 100 and 1000 jobs are considered to evaluate the simulator.

#### Network Topology

The network topology in this work considers 20 hosts, each of them consisting of 1 processing element (PE) of 1500 MIPS. In these hosts, 20 VMs are created, each one with a performance of 1000 MIPS. It must be underlined that a main goal here is to validate the performance of the DVFS algorithm in the simulator, and thus, the critical parameters are the different frequency index values and the maximum MIPS assigned to machines. The maximum value of performance in MIPS cannot be set to an arbitrary value. A too high value of MIPS would make that, even though the frequency multiplier determined by a dynamic governor is scaled down to its minimum, the utilization would never surpass the utilization threshold and the system would always remain in the lowest frequency, making it not possible to observe the DVFS dynamic behavior. On the contrary, if the value of the maximum MIPS value is set too low, then the frequency multiplier would never be scaled down, as the utilization would always be higher than the utilization threshold and the maximum frequency multiplier would always be applied. With the values indicated before, a balance of these parameters is obtained allowing to evaluate the DVFS behavior with the dynamic governors.

Also, it must highlighted here that all the simulations in this work are conducted considering Intel (R) Core (TM) 2 Quad CPU Q6700@2.66 GHz with 4 GB of RAM memory hosts, with the power model for the processing cost (Eq 2) using the real measured values of *P*_*Idle*_ and *P*_*Full*_ for each frequency (Table 1), and that the models for the different DVFS governors follow the design validated in [29]. Hence, since the belonging of jobs to a determined workflow (as in the presented simulator) does not change its processing within a host, the computing power cost and the behavior of the different DVFS governors in the proposed simulator have been validated using a real system and further details can be found in [29].

### Utilization of machine, frequency index and computing power evolution over time

This section analyses the evolution through time of the three main parameters in the DVFS computing model: utilization of machine, frequency index and computing power consumption. The purpose is to justify the right performance of the DVFS strategy in the simulator. To avoid a large number of data for the presented graphs, Montage workflow with 25 jobs is considered in this section to show the behavior of the DVFS strategy. Since the use of a static governor does not involve any adjustments in the frequency (or frequency index and multiplier) when the predefined utilization thresholds are exceeded, consumed computing power only varies with the utilization of the machine following Eq 2, with fixed values for *P*_*full*_ and *P*_*idle*_ and a constant value for utilization of machine and frequency is obtained. In this way, in order to show the dynamic adjustment of the frequency in the simulator, dynamic governors performance is analyzed. Specifically, since OnDemand and Conservative governors essentially differs in the considered criteria for the selection of thresholds, OnDemand governor is analyzed.

**Fig 7:**
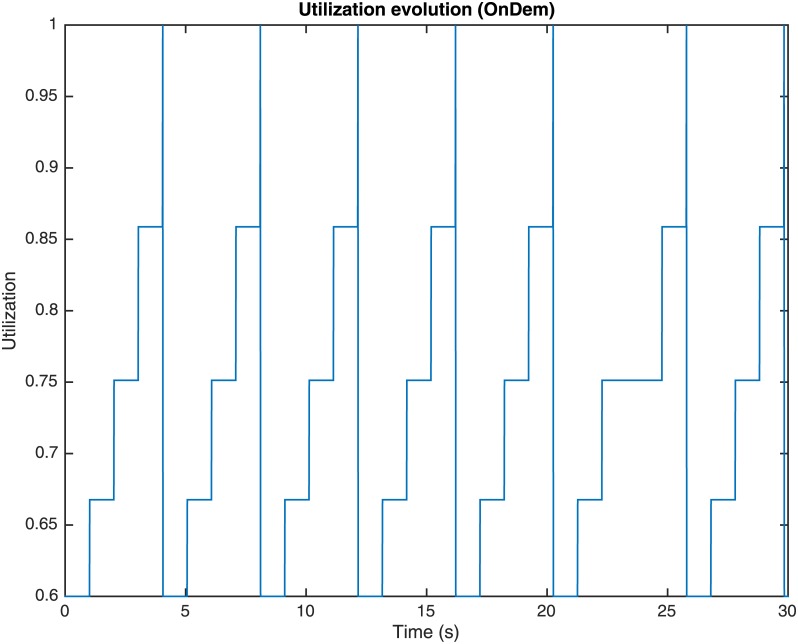
Processing element utilization evolution through time with OnDemand governor.

**Fig 8:**
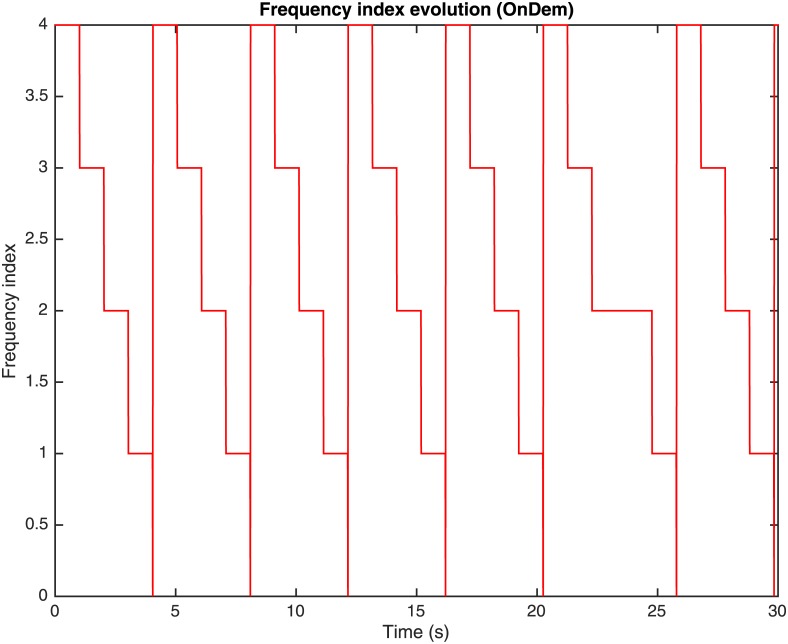
Frequency index evolution through time with OnDemand governor.

**Fig 9:**
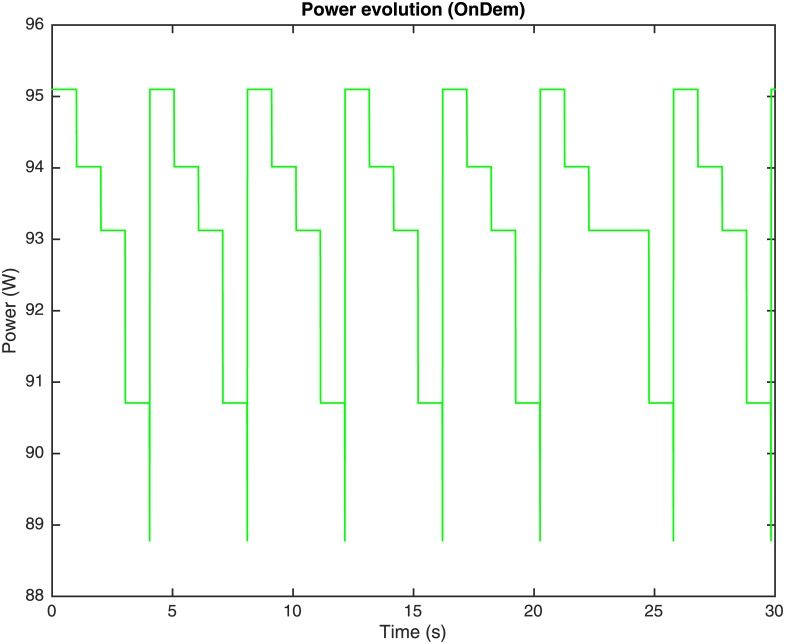
Computing power evolution through time with OnDemand governor.

The performance of the OnDemand governor depends on two configuration parameters: the utilization threshold *α*_*th*_ and the sampling down factor *s*_*down*_. The utilization threshold *α*_*th*_ is the value of utilization *α* which, if surpassed by excess or default, makes the governor change the working frequency of the processing element (or, equivalently, the frequency index and multiplier) and so forth, the values for *P*_*full*_ and *P*_*idle*_ and performance in MIPS. The sampling down factor *s*_*down*_ represents a number of iterations or utilization checking time interval that the governor must wait to scale the working frequency down to the following lower frequency. Whenever the utilization of the processing element *α* surpasses by excess the utilization threshold *α*_*th*_ (i.e., *α* > *α*_*th*_), the governor decides that the performance of the processing element should be increased to accelerate the processing of tasks, and it sets the processor frequency (or, equivalently, the frequency index and multiplier) to the highest value, so that the performance in MIPS is set to its maximum. Once the processing element reaches the highest performance rate, the governor waits *s*_*down*_ iterations to check again its utilization. If the utilization is lower than *α*_*th*_, the working frequency descends one level. This process is repeated until the utilization *α* is increased over the threshold *α*_*th*_, what increments the processor frequency (or, equivalently, the frequency index and multiplier) to its maximum once again. Once the processing element has reached the highest performance rate, the frequency drop process is restarted. Figs 7, 8 and 9, represent the utilization of the processing element *α*, the frequency index and the consumed computing power *P*_*total*_ during the simulation using an OnDemand governor, respectively.

In the simulations corresponding to Figs 7, 8 and 9, *α*_*th*_ = 0.95 and *s*_*down*_ = 100 iterations. As it can be observed in Figs 7 and 8, at the beginning of the simulation the utilization of the processing element is *α* = 0.6 and the frequency index is 4 (corresponding to 2.670 GHz as indicated in Table 1) what corresponds to a power consumption, as shown in Fig 9, that has been calculated as:

$$\begin{array}{c} P_{total}\left(\alpha ,V,f\right)=P_{idle}\left(V,f\right)+\left[P_{full}\left(V,f\right)-P_{idle}\left(V,f\right)\right]\alpha =83.25W+\left[103.00W-83.25W\right]0.6=95.1W \end{array}$$ (6)

where the values for *P*_*idle*_ and *P*_*full*_ are obtained from Table 1. In the following seconds of the simulation, it can be observed that the utilization of the processing element *α* increases gradually (see Fig 7) until *α*_*th*_ is surpassed by excess. In a parallel way, meanwhile *α* < *α*_*th*_ the utilization of the processing element *α* is checked every *s*_*down*_ iterations and given that *α*_*th*_ is not exceeded, the frequency index is scaled down to the following lower value gradually (i.e., 3, 2, 1 and 0) and so, the power consumption decreases, as the values for *P*_*idle*_ and *P*_*full*_ are reduced accordingly to the frequency index, as presented in Table 1. Once the utilization *α* is greater than *α*_*th*_ when it is checked, the frequency index is scaled up to the maximum again (i.e., 4 corresponding to a 2.670 GHz frequency, Fig 8) and the power consumption increases as it can be observed in Fig 9. Hence, it is shown how at the beginning of the simulation the frequency index was set to its maximum (i.e., 4) and that the current load of jobs means an utilization of 0.6, and power of 95.1 W. After 100 iterations, the utilization is checked and as its value is just 0.6, which is lower than 0.95, the frequency index is reduced to 3. The process is repeated until the frequency index is reduced to 1, when the utilization is increased over 0.95 and the DVFS governor sets the multiplier back to 4. As it has been shown, the frequency indexes are set to their maximum whenever the utilization exceeds the threshold *α*_*th*_, and scaled down to save power when it is below, in a gradual way. This method to scale the consumption and performance of the processing elements using OnDemand governor can be considered a double-edged sword. Higher frequencies means more MIPS, what ensures that there is no an unnecessary delay in the jobs execution, but the power consumption is higher, what could be inefficient if the utilization is not high enough. Analogously, a lower value of the frequency reduces power, but the governor must be sure that the utilization rate does not reach 1 with a low frequency index, as that would mean that the jobs would be delayed unnecessarily.

### Time, power and energy global analysis

The implemented governors, Performance, PowerSave, OnDemand and Conservative, are tested processing the twelve different workloads presented in the scenario description: Montage (25, 50, 100 and 1000 jobs), Inspiral (30, 50, 100 and 1000 jobs) and Sipht (30, 60, 100 and 1000 jobs) workflows. The UserSpace governor is not used in the comparison, as its behavior depends on a arbitrary frequency selection by the user. Specifically, four parameters are analyzed to discuss the benefits of each of the DVFS governors: time, overall power consumption, average power consumption and energy. Furthermore, the results of the implemented DVFS governors with a static data center scheduling model (i.e., only DVFS intra-host strategies for energy saving are introduced) are compared to those of the governors when the scheduling in the data center considers a DVFS-based scheduling strategy for energy optimization (i.e., both DVFS intra-host and DVFS inter-host strategies are considered for energy saving simultaneously). As DVFS-based scheduling strategy, the recent and successfull adaptive online energy-aware scheduling strategy presented in [10–12] is implemented, which constitutes an updated version of [13]. This scheduler is based on a computing-plus-communication optimization model which tries to minimize on a per-job basis the overall resulting processing energy. The computing-plus-communication strategy can be formally expressed as:

$$\begin{array}{c} \min\sum_{i=1}^{M}\varepsilon_{CPU}\left(i\right)+\sum_{i=1}^{M}\varepsilon_{Reconf}\left(i\right)+\sum_{i=1}^{M}\varepsilon_{net}\left(i\right) \end{array}$$ (7)

where *ε*_*CPU*_(*i*), *ε*_*Reconf*_(*i*) and *ε*_*net*_(*i*) are the energy values associated to the computing, the frequency reconfiguration and the communication costs, respectively, of the *i*^*th*^ virtual machine *VM*(*i*) with *i* = 1, …, *M* being *M* the total number of virtual machines. The computational cost *ε*_*CPU*_(*i*) is defined as:

$$\begin{array}{c} \varepsilon_{CPU}\left(i\right)=P_{i,total}\left(\alpha ,V,f\right)\cdot t\left(i\right) \end{array}$$ (8)

with *P*_*i*, *total*_(*α*, *V*, *f*) represents the power of virtual machine *VM*(*i*) as explained in Eq 2 and *t*(*i*) is the time when the *VM*(*i*) operates with power *P*_*i*, *total*_(*α*, *V*, *f*). The frequency reconfiguration cost *ε*_*Reconf*_(*i*) represents the cost of changing the switching among discrete frequencies of *VM*(*i*). Two costs must be considered: internal switching cost and external switching cost. The first one is the cost of changing the internal-switching among discrete frequencies of *VM*(*i*) from *f*_*j*_(*i*) to *f*_*j*+*k*_(*i*) where *j* is the number of the discrete frequency in the range *j* = 0, …, *Q* and *k* represents the number of steps movements to reach the next active discrete frequency of the *K* possible, *k* = 1, …, *K*. The second one is the cost for external-switching from the final active discrete frequency of *VM*(*i*) at the end of a job to the next incoming job. The reconfiguration cost is defined as the sum of these two terms:

$$\begin{array}{c} \varepsilon_{Reconf}\left(i\right)=k_{e}\sum_{k=0}^{K}{\left(\Delta f_{k}\left(i\right)\right)}^{2}+k_{e}ExtCost \end{array}$$ (9)

being *k*_*e*_[*Joules*/*Hz*^2^] the reconfiguration cost induced by a unit size frequency switching, Δ*f*_*k*_(*i*) = *f*_*k*+1_(*i*) − *f*_*k*_(*i*), and *ExtCost* the quadratic difference between the last active discrete frequency of *VM*(*i*) for the current job and the first active discrete frequency of *VM*(*i*) in the next incoming job. Finally, the communication cost *ε*_*net*_(*i*) can be expressed as:

$$\begin{array}{c} \varepsilon_{net}\left(i\right)=P^{net}\left(i\right)\sum_{j=1}^{Q}\left(\frac{F_{j}\left(i\right)t_{j}\left(i\right)}{R(i)}\right) \end{array}$$ (10)

where *P*^*net*^(*i*) is the power consumed by the *i*^*th*^ end-to-end connection, *F*_*j*_(*i*) is the *j*^*th*^ processing rate of *VM*(*i*), *R*(*i*) is the communication rate of the *i*^*th*^ end-to-end connection and *t*_*j*_(*i*) is the computing time of *VM*(*i*) working at *F*_*j*_(*i*). Also, *P*^*net*^(*i*) is defined as:

$$\begin{array}{c} P^{net}\left(i\right)=\zeta_{i}\left(2^{R\left(i\right)/W_{i}}-1\right)+P^{idle}\left(i\right) \end{array}$$ (11)

with $\zeta_{i}=\frac{N_{0}\left(i\right)W_{i}}{g_{i}}$, *i* = 1, …, *M*, where *N*_0_(*i*), *W*_*i*_ and *g*_*i*_ are noise spectral power density, transmission bandwidth and (nonnegative) gain of the *i*^*th*^ link, respectively, and *P*^*idle*^(*i*) is the power consumed by the *i*^*th*^ end-to-end connection in the idle mode. Accordingly, the power model in the simulation offering the energy-related values also takes into account the frequency reconfiguration and the communication costs in the final results beyond the computational cost. Hence, the total consumed energy *ε*_*tot*_ in the simulator is calculated as:

$$\begin{array}{c} \varepsilon_{tot}=\sum_{i=1}^{M}\varepsilon_{CPU}\left(i\right)+\sum_{i=1}^{M}\varepsilon_{Reconf}\left(i\right)+\sum_{i=1}^{M}\varepsilon_{net}\left(i\right) \end{array}$$ (12)

The default values of the power model and DVFS-based scheduler for the simulation are presented in Table 2:

**Table 2:** Values of the power model and DVFS-based scheduler main parameters for the simulations.

Parameter	Value
*M*	20
*Q* + 1	5
*R*(*i*)	15 [*Mbps*]
*ζ*_*i*_	0.5 [*mWatt*]
*W*(*i*)	25 [*MHz*]
*k*_*e*_	0.05 [*Joule*/*GHz*^2^]

**Fig 10:**
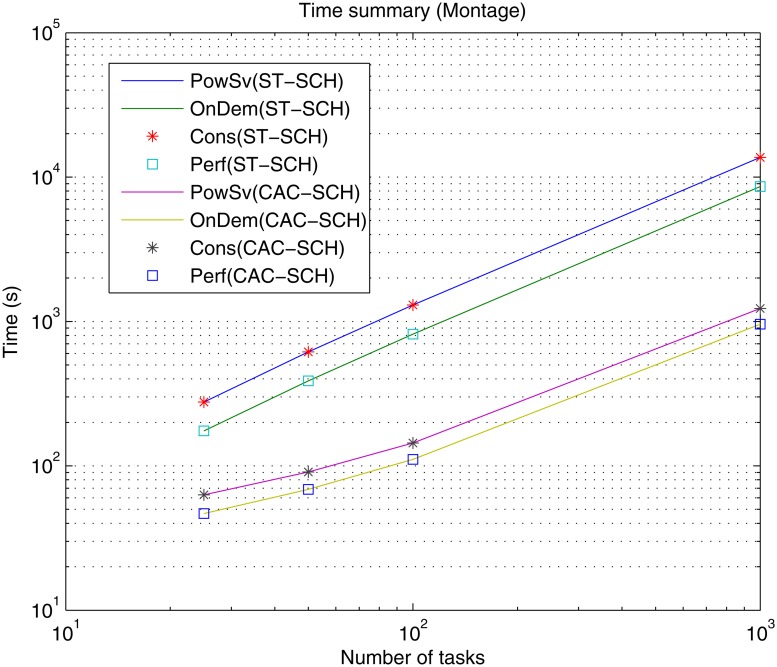
Time (makespan) summary for the different DVFS governors (Perf, PowSv, OnDem and Cons) and the different scheduling strategies (ST-SCH and CAC-SCH) with Montage traces (25, 50, 100 and 1000 jobs).

**Fig 11:**
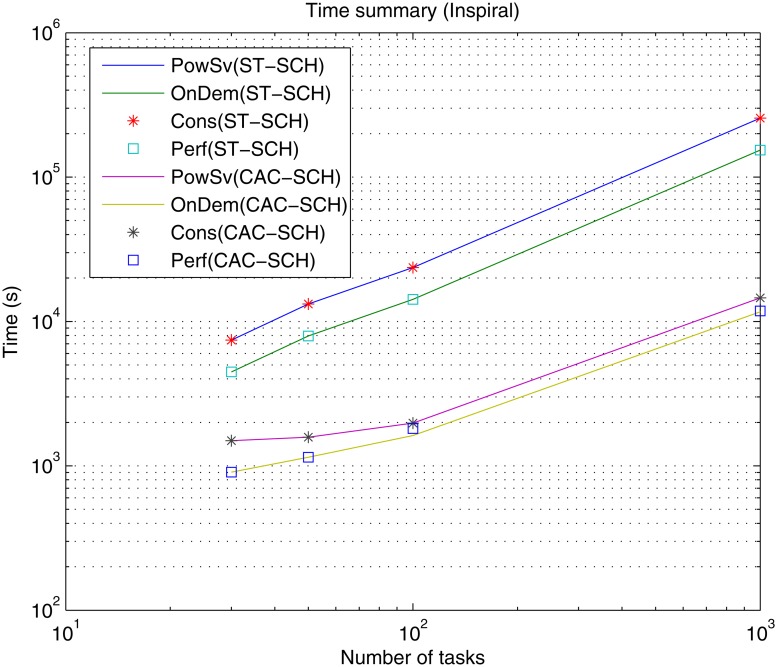
Time (makespan) summary for the different DVFS governors (Perf, PowSv, OnDem and Cons) and the different scheduling strategies (ST-SCH and CAC-SCH) with Inspiral traces (30, 50, 100 and 1000 jobs).

**Fig 12:**
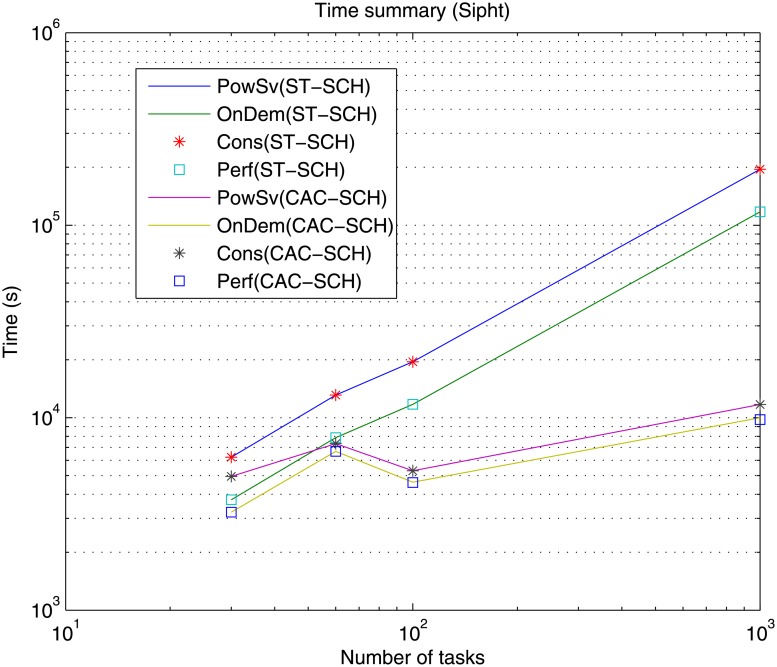
Time (makespan) summary for the different DVFS governors (Perf, PowSv, OnDem and Cons) and the different scheduling strategies (ST-SCH and CAC-SCH) with Sipht traces (30, 60, 100 and 1000 jobs).

Firstly, processing time or makespan results of the simulations are presented. Figs 10, 11 and 12 present makespan results for the different governors, i.e., Performance (Perf), PowerSave (PowSv), OnDemand (OnDem) and Conservative (Cons) and the different scheduling strategies, i.e., static (ST-SCH) and the recent DVFS-based computing-plus-communication strategy (CAC-SHC) presented above [10–12], in the data center for the twelve different workloads, i.e., Montage workflows (25, 50, 100 and 1000 jobs), Inspiral workflows (30, 50, 100 and 1000 jobs) and Sipht workflows (30, 60, 100 and 1000 jobs) are presented.

As expected, it can be observed that, generally, the processing time of the workflows is incremented as the workflow considers a greater amount of jobs with independence of the type of workflow, i.e., Montage, Inspiral and Sipht, type of governor, i.e., Perf, PowSv, OnDem and Cons, and type of scheduling strategy, i.e., ST-SCH or CAC-SHC. However, since the jobs in the workflows are heterogeneous and present different types of dependencies in each case and their processing depend on the considered scheduling strategy, there are workflows with a shorter number of jobs whose processing is larger than other workflows composed of a greater number of jobs. This is the case of the processing of the Sipht workflow involving 60 jobs, whose processing time is larger than that of the Sipht workflow with 100 jobs for the CAC-SCH strategies, as observed in Fig 12. Furthermore, it is shown that makespan results for governors can be categorized into two different types depending on the kind of scheduling strategy in the data center. Specifically, it can be observed that the overall processing time when DVFS governors are the only strategies for energy-optimization in the data center (i.e., the only energy saving mechanisms are the DVFS governors and a static scheduling strategy or ST-SCH scheduling type is considered) is significantly longer than that of provided by the data center when not only the DVFS governors are the energy saving strategies but the workload is scheduled considering a DVFS-based strategy (i.e., an inter-host energy overlapped strategy is added or CAC-SCH scheduling strategy). Also, it is shown, that the dynamic OnDemand governor and the static Performance governor process all workflows in a similar time, which is shorter than that offered by the PowerSave and Conservative governors for every type and number of jobs of the workflows and scheduling strategy. On the one hand, OnDemand governor scales up or down frequency of its associated processing element accordingly to the utilization level. Hence, in conditions of high workload (i.e., when the predefined utilization threshold of the governor is exceeded) this governor scales up the frequency of the processing element in a way that its processing capabilities are increased to execute workload as fast as possible, what is translated in more efficient results in terms of time. On the other hand, Performance governor keeps the frequency of the processing element in the higher value during its whole simulation and thus, the processing element offers the highest processing capabilities though all the workload execution and it is able to offer efficient results in terms of makespan. However, in the case of PowerSave, the governor keeps the frequency of the processing elements in its lowest value, i.e., lowest processing capabilities, what can delay the execution of workload depending on the amount of jobs to be processed. Also, in the case of Conservative governor, the strong gradual adaptation to changing workload conditions can also delay the execution. In this way, it can be appreciated that the time difference between the data center using PowerSave and Conservative governors and the data center using the rest of governors generally grows with the number of jobs involved in the workflow and the greatest difference is found for Montage, Sipht and Inspiral workflows with 1000 jobs (please note the use of a logarithm scale). These results are consistent both considering the static and computing-plus-communication DVFS-based scheduling strategies, although shorter makespan are offered with a dynamic scheduler. Hence, it can be appreciated that OnDemand and Performance governors offer the shortest execution time and that using a DVFS-based scheduling strategy significantly improve these results.

**Fig 13:**
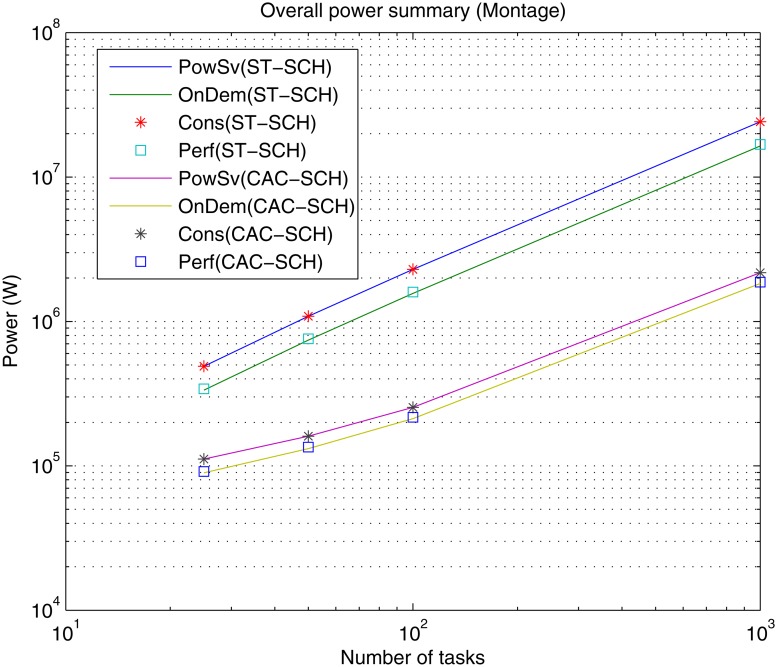
Overall power summary for the different DVFS governors (Perf, PowSv, OnDem and Cons) and the different scheduling strategies (ST-SCH and CAC-SCH) with Montage traces (25, 50, 100 and 1000 jobs).

**Fig 14:**
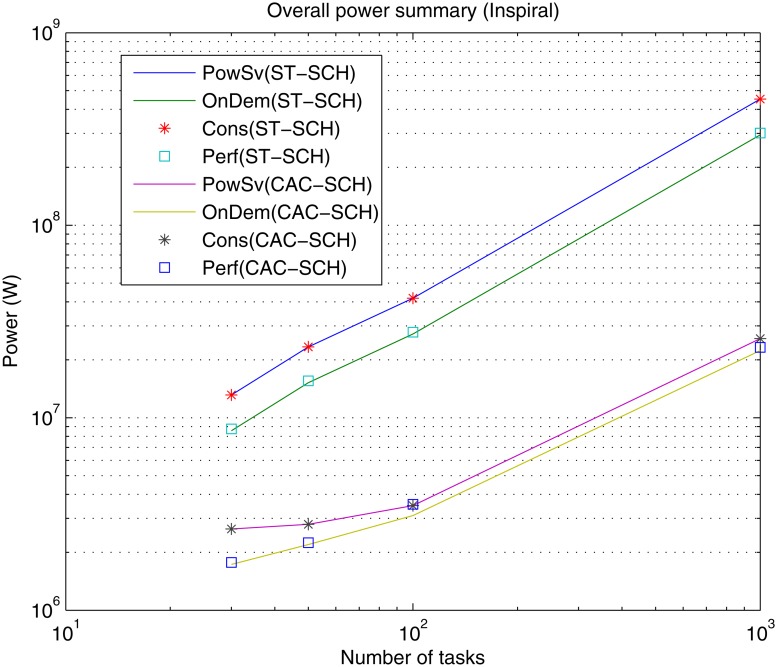
Overall power summary for the different DVFS governors (Perf, PowSv, OnDem and Cons) and the different scheduling strategies (ST-SCH and CAC-SCH) with Inspiral traces (30, 50, 100 and 1000 jobs).

**Fig 15:**
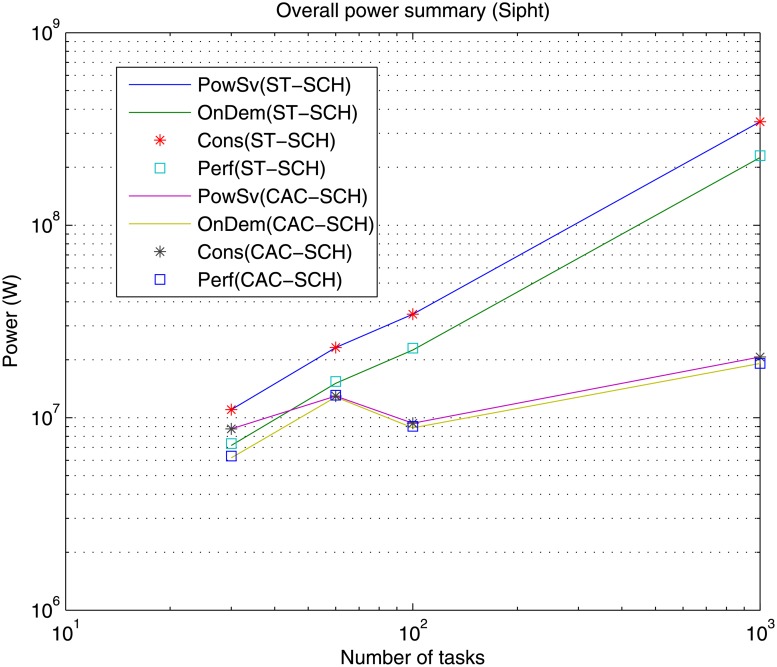
Overall power summary for the different DVFS governors (Perf, PowSv, OnDem and Cons) and the different scheduling strategies (ST-SCH and CAC-SCH) with Sipht traces (30, 60, 100 and 1000 jobs).

Also, Figs 13, 14 and 15 show the overall power needed for the data center to process the different workflows, implemented governors and scheduling strategies.

As in the case of makespan, the overall power grows as the number of jobs of the workload to be processed increases for all types of governors and scheduling strategies for most workflows (with the exception of Sipht with 60 jobs workload processed with CAC-SCH, Fig 15). This represents an expected result since the processing of a greater amount of jobs generally represents a greater power consumption. However, again, since there does not exist homogeneity in the characteristics and dependencies of jobs in the workflows and the way in which they are scheduled, the processing of a higher number of jobs does not necessarily involve a higher power consumption. Additionally, it can be observed that in the case of OnDemand and Performance governors, the overall required power is similar and lower than in the case of PowerSave and Conservative governors. Once again this is a logical result given that OnDemand governor performs a fast adaptation of its power consumption to the current utilization of the processing elements. Hence, OnDemand governor changes its performance accordingly to the current workload conditions, in contrast to PowerSave governor, which keeps its power consumption fixed during the whole execution to the lowest level without considering the utilization rate of the processing elements or Conservative governor, which also performs an adaptation to workload conditions but much more gradually, and so, slow. Also, as shown, Performance governor presents similar results to OnDemand governor in terms of power consumption through the consideration of the highest frequency level during the whole execution, which in the case of high utilization rates results significantly effective. Moreover, as it can be observed, the overall power consumption is lower when the considered scheduling strategy is the computing-plus-communication DVFS-based scheduling strategy, CAC-SCH, since it makes a distribution of workload based on the minimization of power consumption among the different virtual machines.

Also, the average power for the different governors, workloads and scheduling strategies can be considered. The average power in every case is calculated dividing the overall power consumption by the corresponding makespan, and so, it offers a normalized power result. In this case, as expected, a constant value is obtained: 17.6606 W for PowerSave, 19.1460 W for OnDemand, 17.6605 W for Conservative and 19.5781 W for Performance. It is appreciated that the governors offering the lower average power are PowerSave and Conservative. This could be expected, since PowerSave is designed to consume the minimum power during the whole performance of the processing element although this represents, as shown in Figs 10, 11 and 12, larger processing times. Also, in the case of Conservative governor, the slow adaptation capability of this dynamic governor increases the processing time as discussed for makespan, and thus, a low average power consumption can be expected. Analogously, Performance governor offers the highest average power consumption as it fixes power consumption of the processing elements to its maximum during the whole execution. Hence, these results show that the more efficient governor is OnDemand in terms of average power.

Finally, the performance of the different governors must be studied from the energy saving point of view, which results of the multiplication of the consumed energy and the makespan of the processing. Table 3 presents energy results, *ε*_*tot*_, for the implemented governors considering the different schedulers and workload conditions.

**Table 3:** Energy summary (Wh) for the different DVFS governors (Perf, PowSv, OnDem and Cons) and the different scheduling strategies (ST-SCH and CAC-SCH) with Montage traces (25, 50, 100 and 1000 jobs), Inspiral (30, 50, 100 and 1000 jobs) and Sipht (30, 60, 100 and 1000 jobs).

		Perf		PowSv		OnDem		Cons	
Project	Jobs	ST-SCH	CAC-SCH	ST-SCH	CAC-SCH	ST-SCH	CAC-SCH	ST-SCH	CAC-SCH
**Montage**	25	1.96E+01	2.93E+01	1.56E+02	3.48E+01	1.13E+02	2.88E+01	1.56E+02	3.48E+01
	50	3.35E+02	4.84E+01	4.26E+02	5.49E+01	3.30E+02	4.75E+01	4.26E+02	5.49E+01
	100	9.85E+02	8.94E+01	1.18E+03	1.00E+02	9.76E+02	8.80E+01	1.18E+03	1.00E+02
	1000	6.59E+04	9.31E+02	6.80E+04	1.01E+03	6.58E+04	9.20E+02	6.80E+04	1.01E+03
**Inspiral**	30	3.57E+03	6.05E+02	4.79E+03	8.48E+02	3.52E+03	5.95E+02	4.79E+03	8.48E+02
	50	7.82E+03	8.26E+02	9.99E+03	9.78E+02	7.73E+03	8.12E+02	9.99E+03	9.78E+02
	100	2.04E+04	1.85E+03	2.42E+04	1.65E+03	2.02E+04	1.65E+03	2.42E+04	1.65E+03
	1000	1.50E+06	1.49E+04	1.54E+06	1.55E+04	1.50E+06	1.46E+04	1.54E+06	1.55E+04
**Sipht**	30	3.42E+03	1.89E+03	4.44E+03	2.57E+03	3.38E+03	1.86E+03	4.44E+03	2.57E+03
	60	9.24E+03	3.90E+03	1.14E+04	3.90E+03	9.15E+03	3.79E+03	1.14E+04	3.90E+03
	100	1.79E+04	3.00E+03	2.11E+04	3.07E+03	1.77E+04	2.99E+03	2.11E+04	3.07E+03
	1000	1.04E+06	1.20E+04	1.07E+06	1.25E+04	1.04E+06	1.22E+04	1.07E+06	1.25E+04

A gray-scale is used to highlight the quality of the results, where the clearer and darker colors indicate lower and higher energy consumption, respectively. First, it is shown that the energy saving is more significant for those cases in which the communication-plus-communication scheduling DVFS-based strategy in used in the data center (i.e., CAC-SCH). This is an expected result since the performance of the CAC-SCH is based on the minimization of the overall energy consumption on a per-job basis and, as it can be observed, the results are validated with the different types of workload and number of jobs. Additionally, both in the case of static and DVFS-based scheduler, OnDemand governor generally presents the best results, followed by Performance governor. Again, as in the power discussion, similar results are presented for PowerSave and Conservative governors. Also, it is important to note that the difference in energy among the governors significantly increases with the number of jobs in most cases.

Finally, it must be mentioned that the conducted experiments in this work test the simulator performance considering both intra-host strategies, DVFS, and a DVFS-based inter-host strategy, the computing-plus-communication DVFS-based scheduling of the MMGreen framework (i.e., CAC-SCH) [10–12]. However, the consideration of other inter-host strategies for the Cloud management in the resource allocation area can be relevant for further progress in the development of energy-aware strategies in Cloud Computing and important and recent allocations models such as CSAM-IISG [33] could be integrated in the simulator. Furthermore, this simulator could be extended to offer capabilities in Mobile-Edge Computing where energy saving is also a critical problem and many real-world applications are being developed currently [12, 34].

## Conclusions

In this work, a new open source simulator for Cloud Computing energy-aware optimization and analysis for real workflows processing has been presented. The proposal extends the sophisticated WorkflowSim simulator to incorporate a power model allowing the estimation of power consumption in data centers considering the computing, frequency reconfiguration and network costs, and the leading intra-host managing strategy DVFS for dynamic adaptation of voltage and frequency to workload. Five types of DVFS governors are implemented and their performance is evaluated in diverse complex scenarios based on NASA´s Montage, Sipht and Inspiral projects in terms of CPU utilization, frequency scaling, power, energy and time saving. Moreover, the performance of the diverse governors are tested considering a recent DVFS-based scheduling strategy. It can be observed that the OnDemand dynamic governor offers greater energy saving and that this performance is significantly improved when a DVFS-based scheduling strategy is used. Hence, it is shown that the intra-host energy saving strategy of DVFS can be combined with inter-host DVFS-based scheduling strategies to increase energy saving in Cloud Computing. It is intended that WorkflowSim-DVFS platform could be used to develop further inter-host and intra-host energy saving information technology management strategies such as local and meta-scheduling scheduling of jobs and virtual machines considering DVFS on host strategy as backend support, as in updated data centers nowadays. Hence, this work provides an expected tool for future research in the field of Green Cloud Computing where the allocation of jobs with complex dependencies must be considered.

## Supporting Information

S1 File Experimental results for utilization of machine, frequency index and computing power evolution over time. Experimental data corresponding to Figs 7, 8 and 9.
(XLSX)

S2 File Experimental results for time, power and energy global analysis. Experimental data corresponding to Figs 10–15 and Table 3.
(XLSX)

S3 File Experimental results for time, power and energy global analysis in MATLAB format. Experimental data corresponding to Figs 10–15 and Table 3 in MATLAB format.
(MAT)

S4 File WorflowSimDVFS software. Proposed simulator source code, also available at [24].
(ZIP)

## References

[1] Srinivasan S. Cloud Computing Evolution In: Cloud Computing Basics. New York, NY: Springer New York; 2014 p. 1–16. Available from: 10.1007/978-1-4614-7699-3_1.

[2] Kliazovich D, Bouvry P, Granelli F, da Fonseca NLS. Energy Consumption Optimization in Cloud Data Centers. 2015; p. 191–215.

[3] Beloglazov A, Abawajy J, Buyya R. Energy-aware Resource Allocation Heuristics for Efficient Management of Data Centers for Cloud Computing. Future Gener Comput Syst. 2012;28(5):755–768. 10.1016/j.future.2011.04.017

[4] Ma Y, Ma G, Zhang S, Zhou F. Cooling performance of a pump-driven two phase cooling system for free cooling in data centers. Applied Thermal Engineering. 2016;95:143–149. 10.1016/j.applthermaleng.2015.11.002.

[5] Rong H, Zhang H, Xiao S, Li C, Hu C. Optimizing energy consumption for data centers. Renewable and Sustainable Energy Reviews. 2016;58:674–691. 10.1016/j.rser.2015.12.283.

[6] Li K. Power and performance management for parallel computations in clouds and data centers. Journal of Computer and System Sciences. 2016;82(2):174–190. 10.1016/j.jcss.2015.07.001.

[7] Botta A, de Donato W, Persico V, Pescapé A. Integration of Cloud computing and Internet of Things: A survey. Future Generation Computer Systems. 2016;56:684–700. 10.1016/j.future.2015.09.021.

[8] Huang R, Masanet E. Data Center IT Efficiency Measures The Uniform Methods Project: Methods for Determining Energy Efficiency Savings for Specific Measures. 2014;.

[9] Prado RP, Garcia-Galan S, Exposito JEM, Lopez LRL, Rodriguez-Reche R. Processing Astronomical Image Mosaic Workflows With An Expert Broker In Cloud Computing. Image Processing and Communications. 2015;19(4):5–20.

[10] Shojafar M, Canali C, Lancellotti R, Abawajy J. Adaptive Computing-plus-Communication Optimization Framework for Multimedia Processing in Cloud Systems. IEEE Transactions on Cloud Computing. 2016;PP(99):1–1.

[11] Shojafar M, Canali C, Lancellotti R, Abolfazli S. An Energy-aware Scheduling Algorithm in DVFS-enabled Networked Data Centers. In: Proceedings of the 6th International Conference on Cloud Computing and Services Science; 2016. p. 387–397.

[12] Shojafar M, Cordeschi N, Baccarelli E. Energy-efficient Adaptive Resource Management for Real-time Vehicular Cloud Services. IEEE Transactions on Cloud Computing. 2016;PP(99):1–1. 10.1109/TCC.2016.2551747

[13] Cordeschi N, Shojafar M, Baccarelli E. Energy-saving self-configuring networked data centers. Computer Networks. 2013;57(17):3479–3491. 10.1016/j.comnet.2013.08.002.

[14] Mweya CN, Kimera SI, Stanley G, Misinzo G, Mboera LEG. Climate Change Influences Potential Distribution of Infected Aedes aegypti Co-Occurrence with Dengue Epidemics Risk Areas in Tanzania. PLoS ONE. 2016;11(9):1–13. 10.1371/journal.pone.0162649 PMC5040426 27681327

[15] Parker GJ, Leppert T, Anex DS, Hilmer JK, Matsunami N, Baird L, et al Demonstration of Protein-Based Human Identification Using the Hair Shaft Proteome. PLoS ONE. 2016;11(9):1–26. 10.1371/journal.pone.0160653 PMC5014411 27603779

[16] Bharathi S, Chervenak A, Deelman E, Mehta G, Su MH, Vahi K. Characterization of scientific workflows. In: Workflows in Support of Large-Scale Science, 2008. WORKS 2008. Third Workshop on; 2008. p. 1–10.

[17] Montage. http://montage.ipac.caltech.edu;.

[18] Calheiros RN, Ranjan R, Beloglazov A, De Rose CAF, Buyya R. CloudSim: A Toolkit for Modeling and Simulation of Cloud Computing Environments and Evaluation of Resource Provisioning Algorithms. Softw Pract Exper. 2011;41(1):23–50. 10.1002/spe.995

[19] Watanabe EN, Campos PP, Braghetto KR, Macedo Batista D. Energy saving algorithms for workflow scheduling in cloud computing. In: Computer Networks and Distributed Systems (SBRC), 2014 Brazilian Symposium on. IEEE; 2014. p. 9–16.

[20] Alkhanak EN, Lee SP, Rezaei R, Parizi RM. Cost optimization approaches for scientific workflow scheduling in cloud and grid computing: A review, classifications, and open issues. Journal of Systems and Software. 2016;113:1–26. 10.1016/j.jss.2015.11.023.

[21] Abbas A, Loudini M, Grolleau E, Mehdi D, Hidouci WK. A real-time feedback scheduler for environmental energy with discrete voltage-frequency modes. Computer Standards and Interfaces. 2016;44:264–273. 10.1016/j.csi.2015.09.003.

[22] Ibrahim S, Phan TD, Carpen-Amarie A, Chihoub HE, Moise D, Antoniu G. Governing energy consumption in Hadoop through {CPU} frequency scaling: An analysis. Future Generation Computer Systems. 2016;54:219–232. 10.1016/j.future.2015.01.005.

[23] Wang S, Luo B, Shi W, Tiwari D. Application configuration selection for energy-efficient execution on multicore systems. Journal of Parallel and Distributed Computing. 2016;87:43–54. 10.1016/j.jpdc.2015.09.003.

[24] Linares-Cloud-Computing-Research-Group-LinCloud. WorkflowSimDVFS, https://github.com/lin-cloud/WorkflowSimDVFS. 2016;.

[25] Aksanli B, Venkatesh J, Rosing T. Using Datacenter Simulation to Evaluate Green Energy Integration. Computer. 2012;45(9):56–64. http://doi.ieeecomputersociety.org/10.1109/MC.2012.249.

[26] Kliazovich D, Bouvry P, Khan SU. GreenCloud: a packet-level simulator of energy-aware cloud computing data centers. The Journal of Supercomputing. 2010;62(3):1263–1283. 10.1007/s11227-010-0504-1

[27] Chen W, Deelman E. WorkflowSim: A toolkit for simulating scientific workflows in distributed environments. In: E-Science (e-Science), 2012 IEEE 8th International Conference on; 2012. p. 1–8.

[28] Deelman E, Vahi K, Juve G, Rynge M, Callaghan S, Maechling PJ, et al Pegasus, a workflow management system for science automation. Future Generation Computer Systems. 2015;46:17–35. 10.1016/j.future.2014.10.008.

[29] Guérout T, Monteil T, Da Costa G, Neves Calheiros R, Buyya R, Alexandru M. Energy-aware simulation with DVFS. Simulation Modelling Practice and Theory. 2013;vol. 39: pp. 76–91. 10.1016/j.simpat.2013.04.007

[30] Cao F, Zhu MM, Wu CQ. Energy-Efficient Resource Management for Scientific Workflows in Clouds In: Services (SERVICES), 2014 IEEE World Congress on; 2014 p. 402–409.

[31] Buyya R, Beloglazov A, Abawajy JH. Energy-Efficient Management of Data Center Resources for Cloud Computing: A Vision, Architectural Elements, and Open Challenges. CoRR. 2010;.

[32] Juve G, Chervenak A, Deelman E, Bharathi S, Mehta G, Vahi K. Characterizing and profiling scientific workflows. Future Generation Computer Systems. 2013;29(3):682–692. 10.1016/j.future.2012.08.015.

[33] Wei W, Fan X, Song H, Fan X, Yang J. Imperfect Information Dynamic Stackelberg Game Based Resource Allocation Using Hidden Markov for Cloud Computing. IEEE Transactions on Services Computing. 2016;PP(99):1–1.

[34] Yang J, Wang H, Lv Z, Wei W, Song H, Erol-Kantarci M, et al Multimedia recommendation and transmission system based on cloud platform. Future Generation Computer Systems. 2016; p. –. 10.1016/j.future.2016.06.015.

